# Effect of Particle Shape on Mechanical Behaviors of Rocks: A Numerical Study Using Clumped Particle Model

**DOI:** 10.1155/2013/589215

**Published:** 2013-07-25

**Authors:** Guan Rong, Guang Liu, Di Hou, Chuang-bing Zhou

**Affiliations:** ^1^State Key Laboratory of Water Resources and Hydropower Engineering Science, Wuhan University, Wuhan, Hubei 430072, China; ^2^Earth Sciences Division, Lawrence Berkeley National Laboratory, Berkeley, CA 94720, USA; ^3^Key Laboratory of Rock Mechanics in Hydraulic Structural Engineering, Ministry of Education, Wuhan University, Wuhan 430072, China

## Abstract

Since rocks are aggregates of mineral particles, the effect of mineral microstructure on macroscopic mechanical behaviors of rocks is inneglectable. Rock samples of four different particle shapes are established in this study based on clumped particle model, and a sphericity index is used to quantify particle shape. Model parameters for simulation in PFC are obtained by triaxial compression test of quartz sandstone, and simulation of triaxial compression test is then conducted on four rock samples with different particle shapes. It is seen from the results that stress thresholds of rock samples such as crack initiation stress, crack damage stress, and peak stress decrease with the increasing of the sphericity index. The increase of sphericity leads to a drop of elastic modulus and a rise in Poisson ratio, while the decreasing sphericity usually results in the increase of cohesion and internal friction angle. Based on volume change of rock samples during simulation of triaxial compression test, variation of dilation angle with plastic strain is also studied.

## 1. Introduction

Rocks are made from minerals, which are various in chemical composition and crystal morphology. Most Rocks in nature are composed of irregular mineral particles strongly bonded together [1]. The shapes of mineral particles are crucial for mechanical behaviors of rocks. Particle shape has been recognized to affect the mechanics characters of granular material, which has been revealed in several publications [2, 3]. The internal friction angle of different shaped particles was investigated through triaxial compression test by Shinohara et al. [4]. Dodds [5] expounded the influences of particle shape and stiffness effects on soil behavior. Liu et al. [6] quantified particle shapes by digital image method and expounded the correlation of mechanical performance and shape factors defined in their paper. Since various particle shapes in a material, it is very difficult to examine the influence of a specific particle shape. Johanson [7] used plastic pellets of different shapes (round, heart, and stars) coated with soft Tacky Wax to make samples, respectively. Although this method can obtain consistent samples of distinct shape, it is not proper to produce rock or sand samples because of different performance between plastic pellets and these materials.

Many scholars studied mechanical behaviors of granular matter using numerical simulation method [8–10]. Compared with other continuum approaches, the particle mechanics approach based on discrete element method can reproduce the processes of fracture initiation and growth at quasimicro- or macroscopic levels and treat the problems relating to discontinuous large deformation with some simple assumptions and constitutive models [11, 12]. Particle flow codes (*PFC2D* and *PFC3D*) are the most widely used particle mechanics codes [1, 11]. Numerical tests by Kock showed that composition and texture of sediments were relevant to frictional strength and development of shear zone [13]. Furthermore, with the help of *PFC2D*, peak strength, internal friction angle, and thickness of shear zones were also proven to be connected with particle shape [14–16].

However, researches referring to this issue are still not wide and enough; a systematic study to this problem is of urgent demand for understanding mechanism of deformation and failure from quasi-microlevels. And past study results concentrated on materials such as sand or soil, but a few papers discussed how particle shapes in rock affect the mechanical behaviors of rocks [17]. Unlike loose particle materials sand and soil, rock particles are strongly bonded together. The cements in rocks cause different interaction mechanisms between rock particles. Consequently, Potyondy and Cundall [1] proposed a bonded-particle model for rock, where rock is represented by a dense packing of spherical particles that are bonded together. For different particle shapes, there is no a unified quantitative evaluation method due to their complexity. Some results described particle shape mainly based on the qualitative approach, lacking quantitative analysis of particle shapes. 

Hence, the main purpose of this paper is to examine the influences of particle shape on the mechanical behaviors of rocks. From the result of quartz sandstone triaxial compression test and mineral particle shape in quartz sandstone, four representative particle shapes were created. We use sphericity index as the particle shape factor to characterize four representative particles. Then mechanical behaviors of four samples formed by four representative particles were studied, respectively.

## 2. Basic Theory of Particle Flow Method

A general particle flow model simulates the mechanical behavior of materials based on discrete element method. The Newton's laws of motion and the force-displacement law provide the fundamental relationship among force, displacement and particle motion.

Mechanical behaviors of materials are simulated in terms of the movement of each particle and the interparticle forces acting at each contact point. At each contact point, contact behaviors consist of stiffness, slip, and bond [18]. 

In the contact normal direction, the stiffness behavior provides the relation among the contact normal force component *F* ^*n*^, the total normal displacements *U* ^*n*^, and the contact normal stiffness (unit: Pa/m) *K* ^*n*^. In the contact tangential direction, the stiffness behavior relates the increment of shear force Δ*F* ^*s*^, and the increment of shear displacement Δ*U* ^*s*^, as follows:

(1) $$\begin{array}{c} F^{n}=K^{n}U^{n},\\ \Delta F^{s}=-k^{s}\Delta U^{s}, \end{array}$$

where *K* ^*n*^ and *k* ^*s*^ are the contact normal stiffness and tangent stiffness (unit: Pa/m), respectively. In this paper, the linear contact models are adopted, where *K* ^*n*^ and *k* ^*s*^ are independent of displacement. The slip behavior relates slip condition, given by

(2) $$\begin{array}{c} F_{\max}^{s}=\mu \left|F^{n}\right|, \end{array}$$

where *F*_max⁡_^*s*^ is maximum allowable shear contact force and *μ* is the friction coefficient. At every contact point, if |*F* ^*s*^| > *F*_max⁡_^*s*^, then slip is allowed to occur. And during the next calculation cycle, the magnitude of *F* ^*s*^ is set to *F*_max⁡_^*s*^.

The bond behavior acts as a kind of glue joining the two particles. *PFC3D* can simulate not only the loose particle materials such as sand and soil but also the rock-like materials where particles are strongly bonded together [1]. A contact-bond model and a parallel-bonded model are two widely used bond behaviors. Compared with the contact-bonded model, the parallel-bonded model is more suitable for rock, as the parallel-bonded model can transmit both forces and moments at contact point between particles, and this better meets the interaction mechanism of particles. And the stiffness in the parallel-bonded model consists of the contact stiffness and bonding stiffness. In the parallel-bonded model, the bond breakage will cause bonding stiffness inactive, which results in stiffness reduction. In this respect, the performance of stiffness reduction is consistent with experiments where the rock-like materials may failure in either tension or shearing with an associated reduction in stiffness [12, 19]. So the parallel-bonded model is used in this paper.

**Figure 1:**
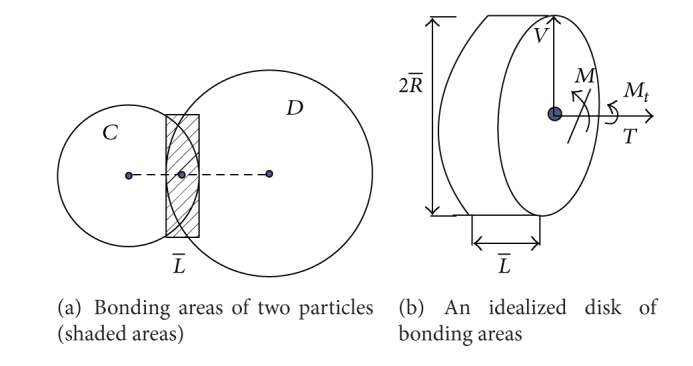
Schematic diagram of parallel bond.

In a 3D model, the bonding areas of two particles with parallel bond are equivalent to a disk (Figure 1(a)). *T*, *V*, *M*, and *M*_*t*_ are tensile force, shear force, bending moment, and twisting moment acted on the bonding cross-section, respectively. The maximum normal stresses *σ*_max⁡_ and shear stresses *τ*_max⁡_ carried by the bonding material can be written as [18]

(3) $$\begin{array}{c} \sigma_{\max}=\frac{T}{A}+\frac{\left|M\right|}{I}\overset{-}{R},\\ \tau_{\max}=\frac{V}{A}+\frac{\left|M_{t}\right|}{J}\overset{-}{R}, \end{array}$$

where *A* and *I* are the area and moment of inertia, respectively, of the parallel-bond cross-section. And *J* is the polar moment of inertia of the parallel-bond cross section. *R* is the radius of the disk (Figure 1(b)). The parallel-bond breaks when either normal stresses or shear stresses exceed corresponding maximum stresses.

## 3. Generation of Models and Determination of Parameters 

### 3.1. Generation of Representative Particles and Samples

**Figure 2:**
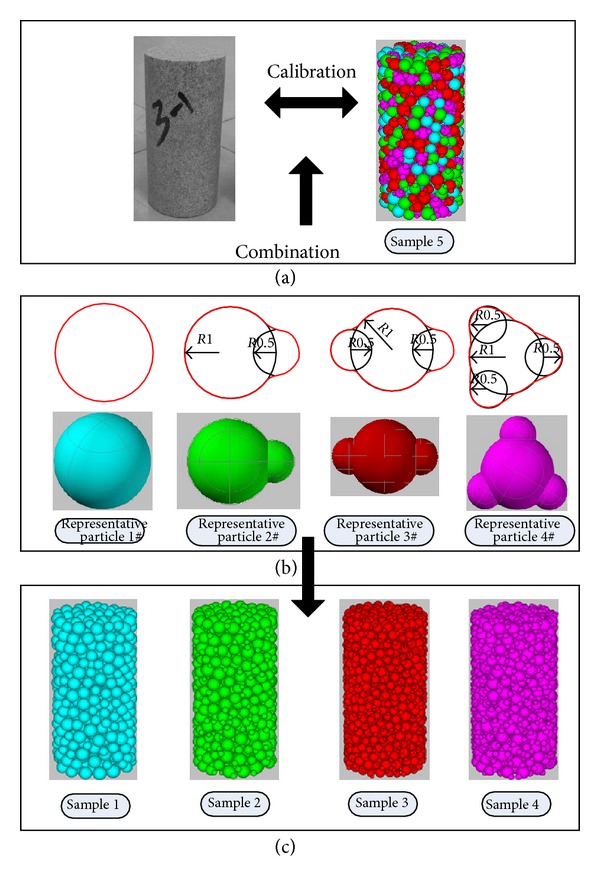
The representative particles and samples.

The mineral particles formed by different morphological structures of crystals in rocks are irregular and often have no fixed shapes. In a numerical simulation of granular material, some representative particles substitute for the real particles [20]. According to the characteristic of quartz sandstone particles, four kinds of representative particles were built to simulate complex particle shapes in quartz sandstone, as shown in Figure 2(b). A common feature of these representative particles adopted in this paper is that they are all made up of a big spherical particle and several small spherical particles. The big spherical particle acts as the main body of representative particles, and the several small spherical particles act as the rugged edges on the surface of rock particles. Figure 2(b) shows the relationship of size between the big spherical particle and the small ones. The samples in Figure 2(c) were formed by four representative particles respectively, and they were used to research mechanical behaviors of different particle shapes. The sample 5 (Figure 2(a)) was created by combinations of the representative particles from 1#–4# to simulate real quartz sandstone.

The representative particle 1# is a ball, which can be created directly in PFC3D. The representative particles 2#–4# can be generated using clumped particles. The clumped particles model is widely used model to generate non-spherical particles [15, 16]. The clumped particles are as a whole consisting of two or more balls in *PFC3D*. The clumped particles can create a group of cement particles that behave as a single rigid body, and these clumped particles may overlap as a deformable body that will not break apart regardless of the forces acting upon it [18].

Sample 1 was first created; then the balls in sample 1 were replaced with representative particles 2#–4#, which produced samples 2–4, respectively. The replacements obey the following three principles [18]. (1) Replace ball with clumped particle, each of which has the same volume as the ball that it replaces. The porosity of sample remains the same after replacement. (2) Each clumped particle is oriented randomly by rotating them about the axes by a random angle. (3) Volume-based centroid of the clumped particle coincides with centroid of the replaced ball.

Considering the generation approach of samples 2–4, the number of balls in sample 4 is four times greater than in sample 1. Although balls in a clumped particle is treated as a single rigid body in calculation cycle, the replacement process mentioned previously will cost too much time if too many balls are generated in the sample 1. To improve calculation speed, we suggest not using too many balls in sample 1. 

### 3.2. Quantitative Description of Particle Shapes

Shape factors to quantify particle shapes are discussed in detail within a number of literatures [6, 21]. Roundness and aspect ratios are very commonly used shape factors [3, 9]. To two-dimensional particles, Kong and Peng [14] suggested to determine shape factors by (4) after analyzing the mechanical behaviors from mesoscopic levels:

(4) $$\begin{array}{c} F=\alpha F_{1}+\beta F_{2},\\ \alpha +\beta =1, \end{array}$$

where *F* is shape parameter, *F*_1_ and *F*_2_ are two parameters related to roundness of particle and roughness of surface, respectively, with *α* and *β* being corresponding weighting coefficients.

The Fourier analysis technique also an important method to characterize particle shapes. The digitized particle outline is described by Fourier analysis which is originally based on the *x-*, *y*-coordinate detection method for contour curves [4, 22].

In this paper, we discuss the mechanical response of three-dimensional particles. Combined with previous shape factors method, considering the feasibility and utility of method, we define sphericity as a shape factors for particles

(5) $$\begin{array}{c} S=\frac{S_{\text{s}}}{S_{p}}, \end{array}$$

where *S*_s_ is surface area of a sphere whose volume is the same with the particle. *S*_*p*_ is the surface area of particle. *S* is sphericity of particle. Equation (5) can be expressed by the volume and surface area of particle, where *V*_*p*_ is the particle volume

(6) $$\begin{array}{c} S=\frac{4\pi }{S_{p}}{\left(\frac{3V_{p}}{4\pi }\right)}^{2/3} \end{array}$$

In order to obtain the volume and surface area of representative particles, four representative particles are created in *AutoCAD*, and their volumes and surface areas are easy to get by *AutoCAD*. Then the sphericities of four representative particles are counted by (4). The result is shown in Table 1. As is shown in Table 1, actually, sphericity expresses the degree of similarity between the sphere and particle. The closer the shape of a particle comes to a sphere, the nearer the sphericity approximates to 1.

**Figure 3:**
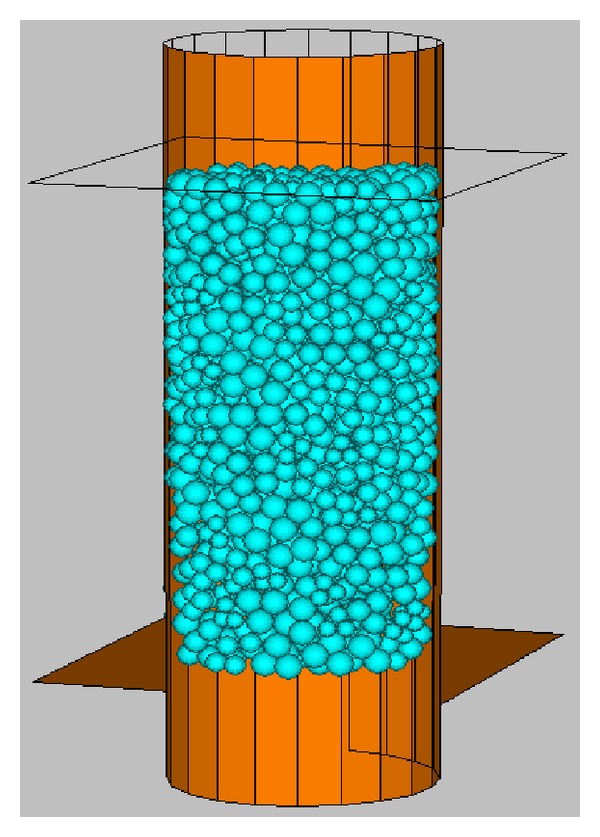
Sample and loading plane.

All the samples are cylinders with height of 80 mm and diameter of 40 mm. The minimal radius of the balls is 2.5 mm, and the ratio of the maximum radius to the minimal radius is 1.5.  Figure 3 shows the location of load planes and a sample. The upper plane and lower plane are two load planes, which load or unload the sample by moving along the axis of the cylinder. The side face of cylinder is a servo plane, which keeps the confining pressure constant by expansion or contraction.

### 3.3. Numerical Calibration of Microscopic Parameters

**Figure 4:**
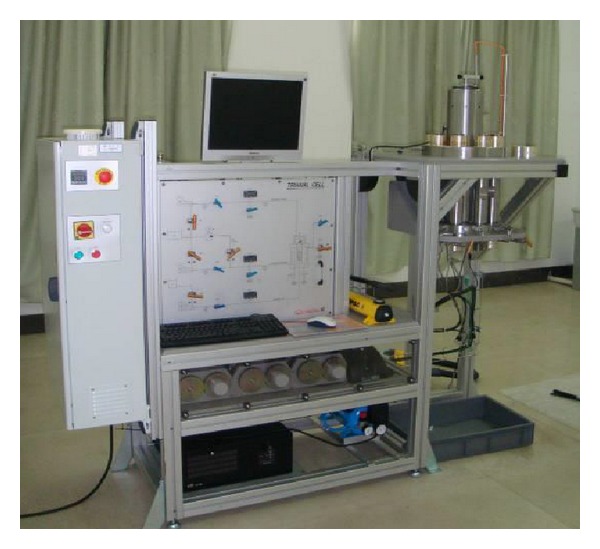
Triaxial coupling test instrument.

Since some microscopic parameters in *PFC* models cannot be obtained directly from laboratory experiments, numerical calibration is required. The microscopic parameters are adjusted to simulate stress-strain curve of quartz sandstone. The quartz sandstone specimens (Figure 4) come from Luojia Mountain, Wuhan, China. For a standard test specimen, test specimen shall be right circular cylinders with a height-to-diameter ratio of 2.0 and a diameter preferable not less than 50 mm. The quartz sandstone specimens used for laboratory test are cylinders having diameter of 50 mm and height of 100 mm. Density of the specimens are 2.65 g/cm^3^. The quartz sandstone specimens are greyish white. The quartz sandstone specimens are composed of 95% quartz clasts, 5% feldspars, and other minerals. Particles sizes are between 0.25–0.50 mm. And the cements are mainly siliceous; the specimens are clastic texture.

**Figure 5:**
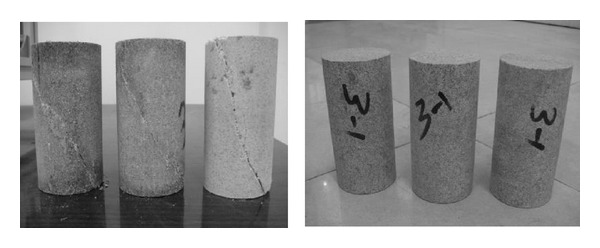
Intact and cracked quartz sandstone samples.

**Figure 6:**
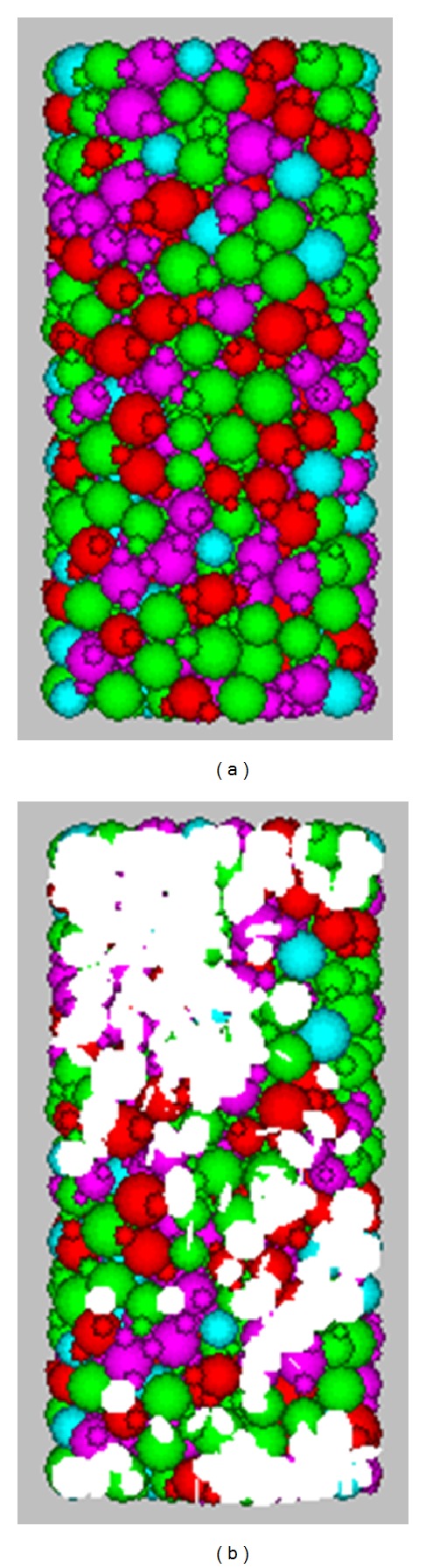
The samples before and during the numerical test.

The triaxial test instrument is researched and developed by the University of Lille and is produced by the company of Top Industria in France (Figure 5). In laboratory experiment, confining pressure is 8 MPa. The quartz sandstone specimens are loaded until destruction, a rupture plane can be seen from Figure 4. Meanwhile, the numerical sample 5 (Figure 6) is used to simulate triaxial compression test in PFC.  Figure 6(a) is sample 5 before the numerical test, and Figure 6(b) shows the distribution of microcracks at the peak stress. The microcracks are showed in white. When a bond breaks either for tension or shearing failure, a new microcrack forms in PFC. From Figure 6(b), we can see the microcracks are concentrated in the diagonal, top, and bottom of the sample. In theory, the microcracks should mainly distribute in the maximum shear stress plane. But in this numerical test, the loads from load planes transmit to the sample only by the contact point of particles and load planes which results in stress concentration easily at the interfaces of sample and load planes. And the bond strength is not homogeneous the standard deviations of bond strengths is showed in Table 2.

In the process of numerical calibration, when the microscopic parameters of sample 5 correspond with the parameters of quartz sandstone, the stress-strain curves both experiment and numerical simulation should be similar (Figure 6).

**Figure 7:**
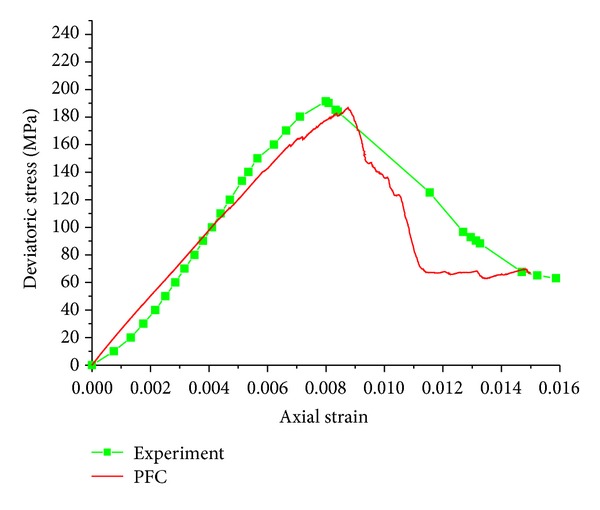
The comparison between numerical test and experiment.

Figure 7 shows the comparison of experiment and numerical simulation. As can be seen in Figure 7, two curves come close before stress get to peak strength. There are slight differences in postpeak region. The main reason is small stiffness of test machine and slow servo response speed of test machine, which cause the large intervals and uneven characteristics of measuring point. So it is reasonable to exist slight differences. 

The agreement between numerical simulation and laboratory results is acceptable (Figure 7), which shows microscopic parameters adopted in the numerical simulation are suitable for the quartz sandstone. The corresponding model parameters are listed in Table 2.

## 4. Results Analysis of Numerical Test

### 4.1. The Influences of Particle Shapes on Strength

To study the relation of particle shapes and mechanical behaviors, numerical triaxial compression test of samples 1–4 (Figure 2) was made under different confining pressures with model parameters listed in Table 2.

**Figure 8:**
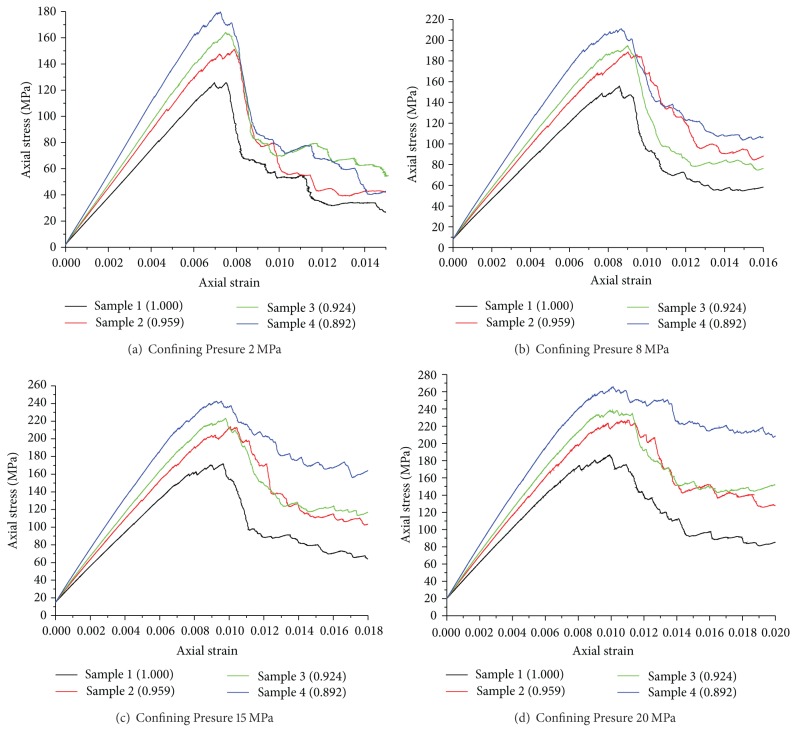
Stress-strain curves of four samples.

Stress-strain curves with confining pressures 2–20 MPa are shown in Figures 8(a)–8(d). The sphericity of particles is noted in parentheses. As can be seen in Figure 8, particle shapes affect stress-strain curve considerably. This impact manifests mainly in the relation between peak strength and particle shapes. Specifically, peak strength decreases with the increasing of sphericity of particles under the same confining pressure. The modulus of elasticity of samples varies with particle shapes, which can be seen from the slope of curves before peak point. The sample with smaller particles sphericity has a higher modulus of elasticity.

Comparing stress-strain curves of different confining pressure (Figures 8(a)–8(d)), we found the influences of particle shapes on residual strength depend on confining pressure. With a high confining pressure and a small sphericity of particles, stress drop at peak point is inconspicuous. Under such condition, the residual strength is close to peak strength. As can be seen from Figure 8(d), when confining pressure maintains a high value (for example 20 MPa), stress drop from peak point is related to sphericity, the smaller the sphericity; the smaller the stress drop. So the residual strength of different samples under high confining pressure varies greatly. While with a low confining pressure, there are little differences about residual strength among different samples. 

At mesoscale level, the results can be explained as follow. Before stress go to peak point, particles bond and interlock together. The smaller the particles sphericity, the greater the degree of interlocking is, correspondingly, the greater the overall peak strength. In the post-peak stage, bonds of particles crack. Particles with big sphericity approaching the spheroid, slip and roll easily, which leads to rapid stress drop. While for small sphericity particles, the effect of interlocking, and friction is stronger, which makes samples remain a higher residual strength in the postpeak stage.

### 4.2. The Influences of Particle Shapes on Crack Initiation Stress and Crack Damage Stress

In the failure process of rock, crack initiation stress and crack damage stress are two important indicators. The crack initiation stress *σ*_ci_ marks crack initiation and stable propagation. When stress exceeds crack damage stress *σ*_cd_, the crack growth is unstable, and *σ*_cd_ is also the beginning of rock dilation.

**Figure 9:**
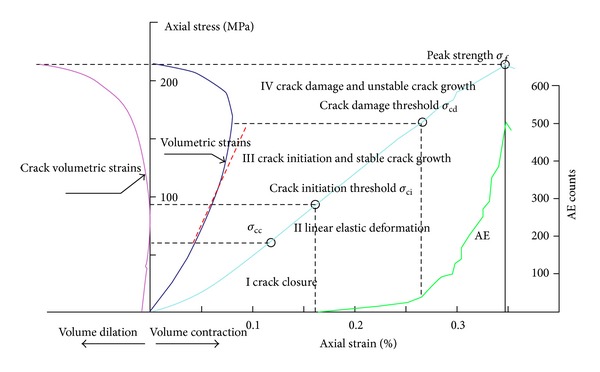
Schematic diagram of stress-strain curves of rocks.

According to the research results of progressive failure process by Martin [23], Eberhardt [24], Eberhardt et al. [25], and Diederichs et al. [26], there are three major ways to ascertain *σ*_ci_, as is shown in Figure 9. (1) Acoustic Emission Test (AE). Eberhardt put out that *σ*_ci_ is the stresses when new AE counts first rise above background. (2) The stress-volumetric strain curves. When stresses get to *σ*_ci_, the stress-volumetric strain curves deviate from the elastic line. (3) Crack volumetric strains. The crack volumetric strains deviate from zero with *σ*_ci_ coming. The crack volumetric strains are defined as (7) by Martin [23]:

(7) $$\begin{array}{c} \varepsilon_{\text{cv}}=\varepsilon_{v}-\frac{\left(1-2\nu \right)\left(\sigma_{1}-\sigma_{3}\right)}{E}, \end{array}$$

where *ε*_cv_ and *ε*_*v*_ are the crack volumetric strains and volumetric strains, respectively, *ν* is poisson ratio, and *E* is elastic modulus.

The crack damage stresses *σ*_cd_ can be ascertained by AE or the stress-volumetric strain curves. When stress reaches *σ*_cd_, AE counts curves show transition and AE counts increase rapidly. In addition, from the stress-volumetric strain curves we can observe volumetric strains rate is near zero and a transition point arises with the crack damage stresses, *σ*_cd_ coming, which are shown in Figure 9.

**Figure 10:**
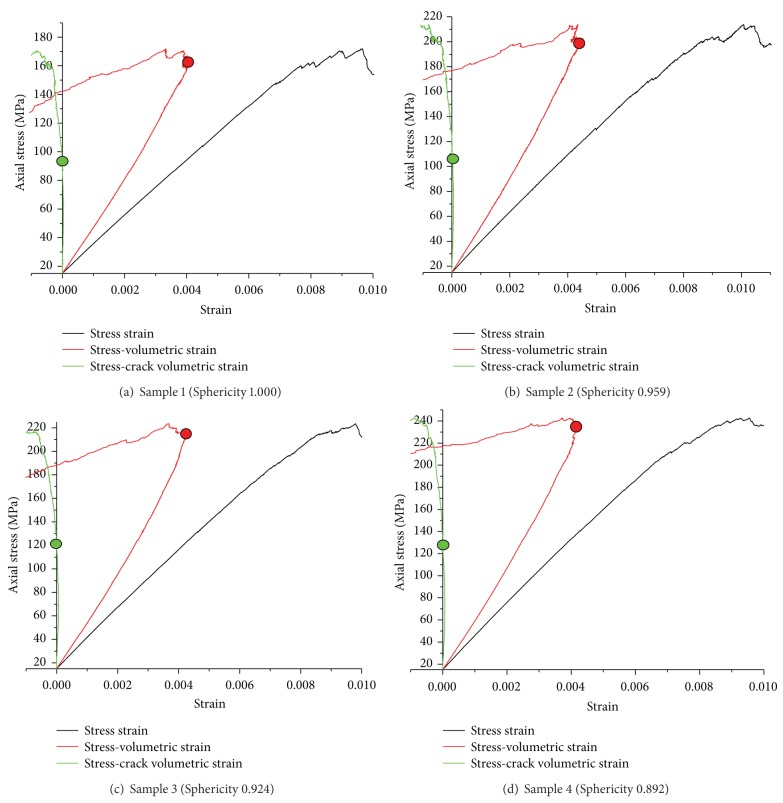
The crack initiation stress and crack damage stress of four rock samples.

This paper determined the crack initiation stress and crack damage stress of rock samples by crack volumetric strains and the stress-volumetric strain curves. Relevant curves of four samples under confining pressure 15 MPa are shown in Figure 10. As mentioned before, the stress that crack volumetric strains deviate from zero is *σ*_ci_, marked with green circle in Figure 10. And the *σ*_cd_ can be obtained from volumetric strain reversal, marked with red circle in Figure 8.

**Figure 11:**
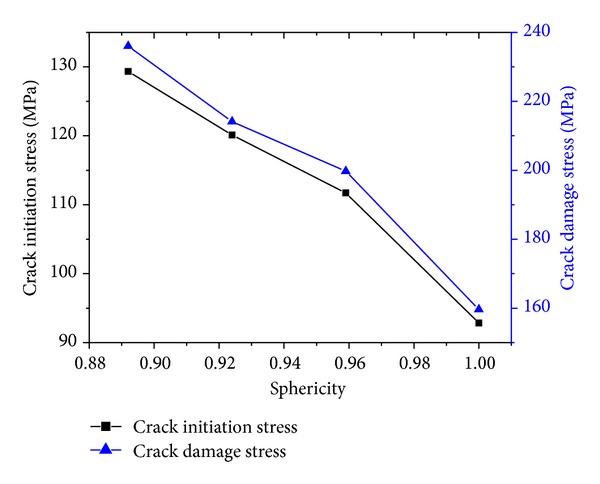
The relation among the crack initiation stress, crack damage stress, and sphericity.

Further, Figure 11 shows how the crack initiation stress and crack damage stress vary with particles sphericity. As we can see from Figure 11, the crack initiation stress and crack damage stress reduce with particles sphericity increasing. In other words, particle shapes affect crack initiation and unstable crack growth in the process of rock failure. With a smaller particles sphericity, rocks can support a higher load before appearing crack initiation and unstable crack growth.

### 4.3. The Influences of Particle Shapes on Cohesion and Internal Friction Angle

Cohesion *c* and internal friction angle *ϕ* are two important material parameters of the Mohr-Coulomb strength theory. The Mohr-Coulomb yield criterion is expressed by (8) using principal stresses. The Mohr-Coulomb yield criterion assumes the cohesion *c* and internal friction angle *ϕ* of rock are both constant, but this assumption is limitation in practical application. Since some scholars [27–29] found cohesion *c* and internal friction angle *ϕ* of rock were not constant in triaxial compression test,

(8) $$\begin{array}{c} \frac{1}{2}\left(\sigma_{1}-\sigma_{3}\right)-\frac{1}{2}\left(\sigma_{1}+\sigma_{3}\right)\sin\phi -c\cos\phi =0, \end{array}$$

where *σ*_1_ and *σ*_3_ are maximum principal stress and minimum principal stress, respectively. Supposing rock samples yield at peak strength, and strength *σ*_*p*_ and confining pressure *σ*_*c*_ are substituted into (8), we get (9):

(9) $$\begin{array}{c} \sigma_{p}=\frac{2c\cos\phi }{1-\sin\phi }+\frac{1+\sin\phi }{1-\sin\phi }\sigma_{c}. \end{array}$$

Equation (9) shows the relation between the peak strength and confining pressure under triaxial condition.

**Figure 12:**
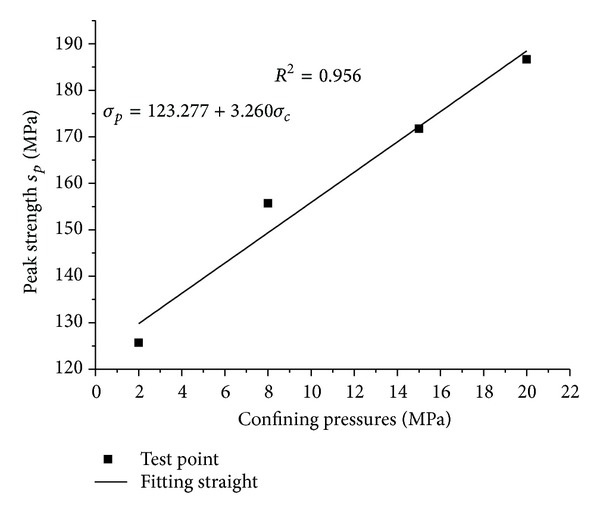
The peak strength versus confining pressure for sample 1.

Based on numerical triaxial compression tests with the confining pressure of 2 MPa, 8 MPa, 15 MP, and 20 MPa, *σ*_*p*_ − *σ*_*c*_ curve of sample 1 is displayed (Figure 12). As is shown by Figure 12, there is a good linear relation between *σ*_*p*_ and *σ*_*c*_. Then *σ*_*p*_ − *σ*_*c*_ curve can be obtained by beeline fitting, which is given in Figure 12. Comparing the linear equation in Figure 12 and (9), cohesion *c* and internal friction angle *ϕ* can be calculated by the gradient *a* and the constant *b* of linear equation (see (10)):

(10) $$\begin{array}{c} \phi =\arcsin\left(\frac{a-1}{a+1}\right),\\ c=\frac{b\left(1-\sin\phi \right)}{2\cos\phi }. \end{array}$$

**Figure 13:**
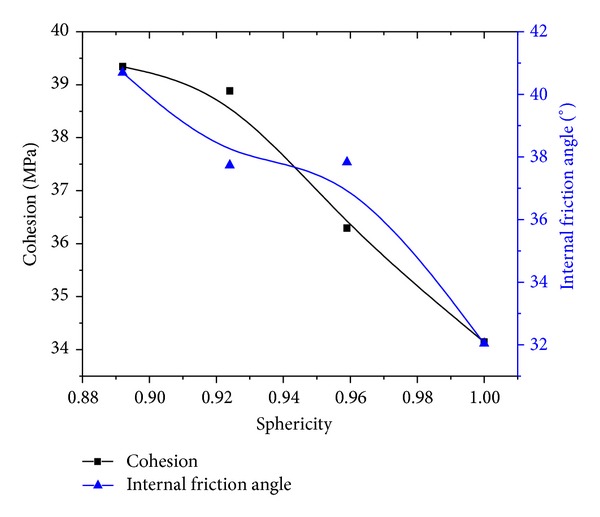
The variation of cohesion and internal friction angle with sphericity.

Along the same ways, cohesion *c* and internal friction angle *ϕ* of other samples are acquired.  Figure 13 shows how cohesion *c* and internal friction angle *ϕ* vary with particles sphericity. As can be found in Figure 13, basically, cohesion *c* and internal friction angle *ϕ* of samples reduce with particles sphericity rising. When particles sphericity is between 0.92 and 0.96, the variation of internal friction angle *ϕ* is gentle.

### 4.4. The Influences of Particle Shapes on Elastic Modulus and Poisson Ratio

Elastic modulus showed a downward trend as particles sphericity rise in Figure 8. To facilitate our analysis, suppose rocks samples are isotropic and compress in the *y*-direction. In numerical triaxial compression tests, elastic modulus *E* and Poisson ratio *ν* can be calculated as follows:

(11) $$\begin{array}{c} E=\frac{\Delta \sigma_{y}}{\Delta \varepsilon_{y}},\\ \nu =-\frac{\Delta \varepsilon_{x}+\Delta \varepsilon_{z}}{2\Delta \varepsilon_{y}}=-\frac{\Delta \varepsilon_{V}-\Delta \varepsilon_{y}}{2\Delta \varepsilon_{y}}, \end{array}$$

where Δ*ε*_*x*_, Δ*ε*_*y*_, and Δ*ε*_*z*_ are strain increments in *x*-, *y*- and *z*-directions, respectively. And Δ*ε*_*V*_ is volumetric strain increment, Δ*σ*_*y*_ is stress increment in *y* direction. Ii is obviously that *E* and *ν* change with load process. But, for simplicity sake, elastic modulus *E* and Poisson ratio *ν* are subject to the point of half peak stress. 

**Figure 14:**
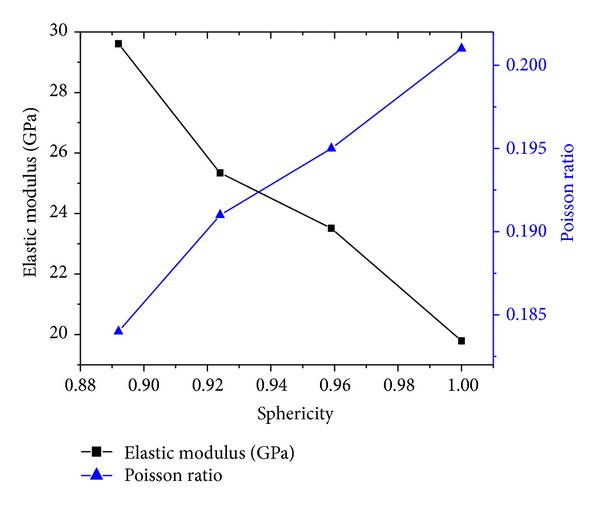
The variation of modulus of elasticity and Poisson ratio with sphericity.

Figure 14 shows how elastic modulus and Poisson ratio vary with particles sphericity under confining pressure 15 MPa. As particles sphericity increase, elastic modulus of samples drops and Poisson ratio rises. As can be seen from the magnitude of Poisson ratio, there are only small differences between Poisson ratio in spite of sphericity influence.

### 4.5. Dilation Effect of Samples

In the process of numerical triaxial compression test, the samples are compressed first, and then they are dilated. In continuum mechanics, dilation angle is the widely used parameter to discribe the dilation effect. Dilation angle is not a constant during the deformation process of rock [30].

But, considering the rock materials may not obey Drucker's stability postulate [31], which recites that the work of the external agency on the displacement produced must be positive or zero, the nonassociated flow rule should be adopted as follows:

(12) $$\begin{array}{c} {\dot{\varepsilon }}_{ij}^{p}=\lambda \frac{\partial g}{\partial \sigma_{ij}}, \end{array}$$

where *λ* is a plastic multiplier and *g* is plastic potential. One of the commonly used plastic potential assumptions states that [32]

(13) $$\begin{array}{c} g=\sigma_{1}-K_{\psi }\left(\sigma_{ij},\eta \right)\sigma_{3},\\ K_{\psi }\left(\sigma_{ij},\eta \right)=\frac{1+\sin\psi \left(\sigma_{ij},\eta \right)}{1-\sin\psi \left(\sigma_{ij},\eta \right)}, \end{array}$$

where *ψ* is dilation angle and *σ*_1_, *σ*_3_ are the maximum and minimum principal stress, respectively. *σ*_*ij*_ is the stress tensor and *η* is the plastic parameter. Here, *η* can be expressed as shear plastic strain, as follows:

(14) $$\begin{array}{c} \eta =\gamma^{p}=\varepsilon_{1}^{p}-\varepsilon_{3}^{p}. \end{array}$$

The dilation angle of rock can be obtained by (15) [33], where ${\dot{\varepsilon }}_{v}^{p}$ and ${\dot{\varepsilon }}_{1}^{p}$ are volumetric and axial plastic strain increments, respectively,

(15) $$\begin{array}{c} \sin\psi =\frac{{\dot{\varepsilon }}_{v}^{p}}{-2{\dot{\varepsilon }}_{1}^{p}+{\dot{\varepsilon }}_{v}^{p}}. \end{array}$$

**Figure 15:**
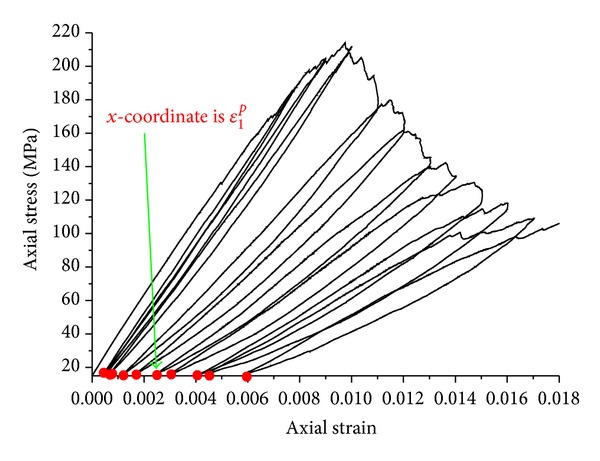
Loading and unloading of sample 2.

Volumetric and axial plastic strain can be obtained by loading and unloading cycles [32, 34]. Since the dilation effect in prepeak stage is not obvious, we focus on evolvement laws of dilation angle in postpeak stage.  Figure 15 shows the stress-strain curve from loading and unloading cycles under confining pressure 15 MPa. The numerical test process is as follows. 

**Figure 16:**
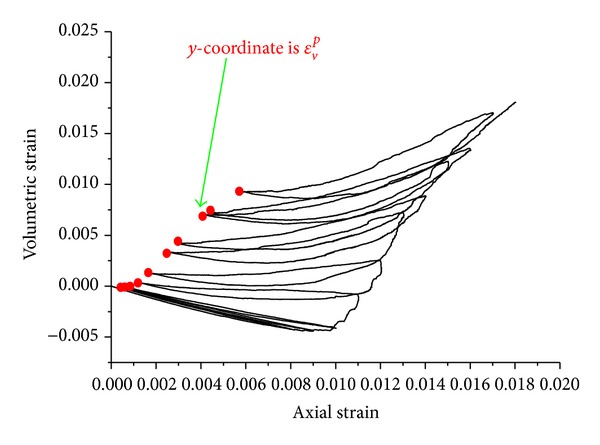
Volume strain curve of sample 2.

The confining pressure and axial stress of 15 MPa are applied on samples first, in which case samples work in elastic behavior. That is plastic strains still do not occur; this state is called original state. Then axial stresses increase to load samples and axial strain is recorded from the original state. When axial strains reach to 0.008, 0.009, 0.0100, 0.011, 0.012, 0.013, 0.014, 0.015, 0.016, and 0.017, unloading, respectively until samples return to the original state. The strains in original state are axial plastic strains *ε*_1_^*p*^, marked with red circle in Figure 15, and then load to the next unloading point, and so forth. In the process of loading and unloading cycle, volumetric strains are recorded, as it is shown in Figure 16. The volumetric strains *ε*_*v*_^*p*^ are marked with red circle in Figure 16.

Equation (15) is conveniently written as incremental form, as follows:

(16) $$\begin{array}{c} \sin\psi =\frac{\Delta \varepsilon_{v}^{p}}{-2\Delta \varepsilon_{1}^{p}+\Delta \varepsilon_{v}^{p}}. \end{array}$$

Given the deformation conditions of triaxial compression test, the following formula is deduced.

(17) $$\begin{array}{c} \varepsilon_{2}^{p}=\varepsilon_{3}^{p},\\ \varepsilon_{v}^{p}=\varepsilon_{1}^{p}+2\varepsilon_{3}^{p}. \end{array}$$

Dilation angles can be calculated according to (16). The volumetric strains *ε*_*v*_^*p*^ and axial plastic strain *ε*_1_^*p*^ can be obtained from loading and unloading cycles in Figures 15 and 16. Shear plastic strain *η* can be calculated using (14) and (17).

**Figure 17:**
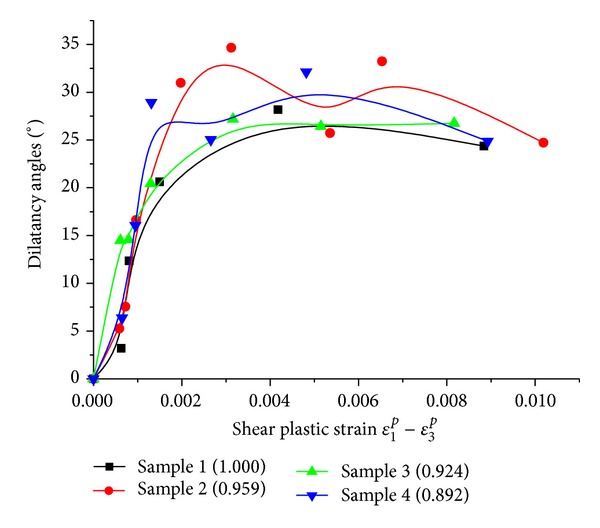
The relation between dilation angle and shear plastic strain.

Figure 17 shows the evolvement process of dilation angle with shear plastic strain. As can be seen from Figure 17, dilation angles increase rapidly in the beginning. Dilation angles reach a maximum around shear plastic strain 0.003. Then, there was no significant change on dilation angles. According to the research of Alejano and Alonso [32] and Zhao and Cai [34], dilation angles will start to fall when shear plastic strain is larger. As shown in Figure 17, although dilation angles of four samples have a little difference, there are no essential distinctions on trends of curves among four samples. That is, particle shapes only impact size of dilation angles rather than trends of curves.

## 5. Discussions

**Figure 18:**
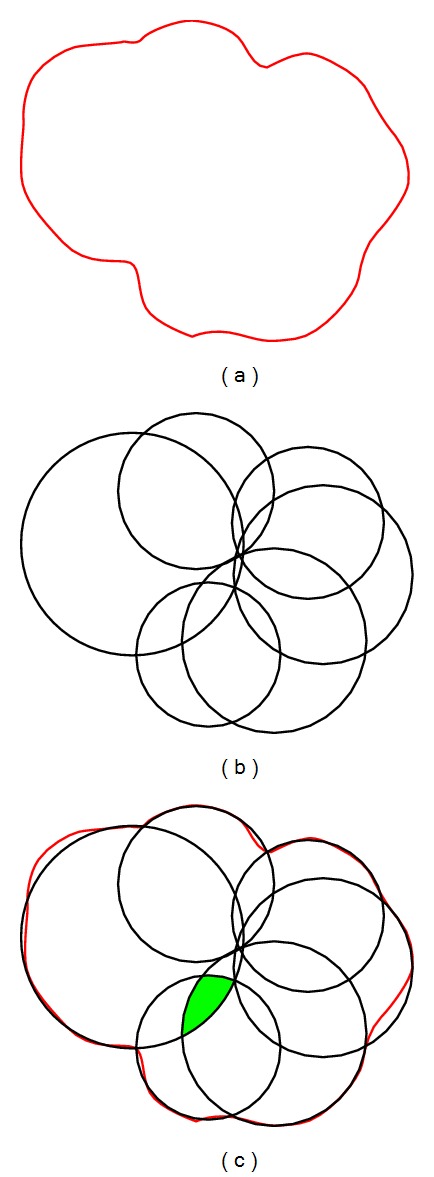
Simulating complex particle shape.

The real shape of mineral particles in rocks is quite complex, while the shape of representative particles used in this paper is simple. Theoretically, the method forming representative particles by clumped particles is also effective for complex particles (Figure 18). The red lines represent the planar contour for a complex particle, and the black lines represent the clumped particles in Figure 18. As is shown in Figure 18(c), the clumped particles can be a good approximation of the complex particle. That is to say, the real shapes of mineral particles in rocks can be simulated using clumped particles in PFC. Deserved to be mentioned, when the overlap parts in a clumped particle involve over two particles (the green part in Figure 18(c)), the clump volume automatically calculated in PFC is inaccurate [18]. In this case, tools such as AutoCAD can be use to compute the clump volume.

The sphericity is not the only shape factor to characterize particle shapes. As a matter of fact, the number and type of representative particles used in this paper are limited. To be more precise, for this kind of representative particles, which are formed by a big spherical particle and several small spherical particles, the sphericity is a relatively efficient shape factor. However, for other kinds of particles, such as elongated particles, aspect ratios and other shape factors may be more effective. It is verified in our study that there are good correlations between the main mechanical parameters of samples and the sphericity of particles. But, the sphericity does not significantly influence the residual strength and dilation effect. And, when particles shapes get more complex, the relation between the sphericity and the internal friction angle becomes unclear. The main reason for these problems may lie in the fact that the sphericity only expresses the degree of similarity between a sphere and particle. In other words, although the sphericity expresses much of morphological characters especially for simple particle shapes, some morphological characters of particle cannot be entirely reflected by the sphericity. So we need to take into account more parameters to further express particle shapes.

## 6. Conclusions

Our numerical experiments show that the mechanical behaviors of rock are influenced by their particle shapes; the primary conclusions are as follows.

1. The sphericity index is an applicable shape factor to measure particles shapes. The sphericity describes the proximity of a particle to a sphere, which directly influences the interlocking ability of particles.
2. The crack initiation stress, crack damage stress, and peak stress of rock are affected by particle shapes. Specifically, three stress indices decrease with increasing of sphericity. The increasing sphericity also leads to smaller elastic modulus and larger poisson ratio. And the decreasing sphericity causes particle interlocking of different degrees which restrains slip and rotation, consequently, cohesion and internal friction angle rise.
3. To samples of different particle shapes, the evolvement process of dilation angle with shear plastic strain was researched. The result shows the trends of dilation angle changing with shear plastic strain are similar, but values of dilation angle have a little difference.

## Figures and Tables

**Table 1:** Shapes factor of representative particles.

Number	1#	2#	3#	4#
Sphericity	1.000	0.959	0.924	0.892

**Table 2:** Microscopic parameters of *PFC3D* model.

Name	Value
Minimum ball radius, *R*_min⁡_ (mm)	2.5
Ball radius ratio, *R*_max⁡_/*R*_min⁡_	1.5
Ball density (Kg/m^3^)	2600
Ball-ball contact Young's modulus (GPa)	25
Young's modulus of parallel bond (GPa)	20
Ball stiffness ratio, *k*_*n*_/*k*_*s*_	2.5
Parallel bond stiffness ratio	2.5
Particle friction coefficient	1.0
Parallel bond normal strength, mean (MPa)	130
Parallel bond normal strength, std. dev (MPa)	10
Parallel bond shear strength, mean (MPa)	130
Parallel bond shear strength, std. dev (MPa)	10
